# Chronic Unexplained Vomiting: A Case Report on Psychogenic Vomiting

**DOI:** 10.7759/cureus.25959

**Published:** 2022-06-15

**Authors:** Gokul Paidi, Marie Jean, Ayobami Oduwole, Nodana Gautam, Karan Kapoor, Ramprasad Paidi

**Affiliations:** 1 Family Medicine, The Medina Clinic, Grandview, USA; 2 Psychiatry, California Institute of Behavioral Neurosciences and Psychology, Fairfield, USA; 3 Family and Community Medicine, The Medina Clinic, Grandview, USA; 4 Medical Education, Weifang Medical University, Weifang, CHN; 5 General Medicine, Aureus University School of Medicine, Oranjestad, ABW; 6 Internal Medicine, Sri Nijalingappa Medical College, Bagalkot, IND

**Keywords:** low mood, psychotherapy, depression, psychogenic vomiting, schizophrenia

## Abstract

Psychogenic vomiting is defined as recurrent vomiting without any known discernible organic reasons, or functional vomiting occurring from psychological stress. The overall clinical presentation of psychogenic vomiting suggests a wide range of disability levels, such as stress disorders, mood changes, depression, sexual problems, personality disorders, learned responses, conversion disorders, and illness problems. Non-pharmacological treatment modalities are important as medication for psychogenic vomiting such as individual and group psychotherapy, supportive and behavioral therapy, and family and marital therapy. In this case report, a 25-year-old male with schizophrenia on medication presented to an outpatient clinic with the primary complaint of recurrent vomiting. After ruling out pathologic and physiologic causes of vomiting. It was classified as psychogenic vomiting.

## Introduction

In medicine, nausea and vomiting can have infectious causes, toxic, iatrogenic, or may be due to gastrointestinal disorders. Sometimes, it can be because of psychiatric conditions [1]. Surprisingly, there is little psychiatric literature on vomiting that may be 'psychogenic in origin' and linked to a psychosocial stressor.

Leibovich defined psychogenic vomiting as recurrent vomiting without any known organic cause, or functional vomiting occurring from psychological stress. The term psychogenic vomiting was first described in the 1960s but lately, it has been termed “functional nausea and vomiting. Psychogenic vomiting occurs as an emotional disturbance and can affect all age groups. Hysterical neurosis or depression was reported by a few authors as the cause of psychogenic vomiting [2]. It may appear benign with few physical discomforts or it can be part of a more complicated and serious disorder such as bulimarexia or anorexia nervosa [3]. Psychiatrists predict the more serious end of this spectrum as serious dehydration problems and severe weight loss. Psychogenic vomiting should be distinguished from chronic idiopathic nausea, cyclical vomiting syndrome, or functional vomiting [4].

From various published literature, a holistic clinical picture of psychogenic vomiting is suggestive of the wide variation in disability degree, it can be a short-lasting minor illness or a disabling and chronic condition. The various psychosocial mechanisms that can be involved are stress disorders, mood variations, depression, sexual issues, personality disorders, learned reactions, conversion disorders, and illness problems [5].

The causative factors, etiology, and pathophysiology of psychogenic vomiting are unclear, and treatment methods and guidelines have not yet been established. This field has recently been highly recognized, but not deeply researched. To rule out the cause, it is emphasized to do a careful evaluation of the patient, other manifestations, duration and frequency, time of the day in which it occurs, stressful events related to vomiting, patient’s ability to verbalize and communicate the problems, to comprehend the treatment plan and expectations from the treatment. Treatment will vary for different patients [6]. Treatment modalities may include pharmacotherapy with the application of the cognitive-behavioral theory approach in addition to supportive hands from the family members to manage the functional symptoms. There is a need for a comprehensive approach to diagnosing and managing patients with functional nausea and vomiting [7].

## Case presentation

A 25-year-old male with a known case of schizophrenia diagnosed a year ago on quetiapine and fluoxetine came to the psychiatry outpatient department (OPD) with a history of frequent and recurrent vomiting. He described his vomiting as sudden in onset without nausea. The vomitus was mostly the food he eats, if he is on an empty stomach the vomitus is clear liquid. The patient even vomits when he eats the foods that he likes the most. The patient denies any aggravating or relieving factors. He described a history of decreased appetite, chest discomfort, predominant irritable mood, decreased attention, weight loss, forgetfulness, suicidal ideation auditory hallucinations, and delusions; auditory hallucinations were self-hurting sounds. He was admitted as an inpatient for vomiting and relapse of his schizophrenia symptoms; the initial laboratory investigations like complete blood count, complete metabolic panel, and electrocardiogram (ECG) were normal. The patient was kept under observation for three days. Temporal association between the start of current antipsychotic fluoxetine and quetiapine was ruled out as the patient tried different antipsychotics before but with similar symptoms of vomiting. The patient was treated symptomatically with omeprazole and ondansetron. The patient’s sleep improved, and vomiting subsided after three days. The patient and his family members were counseled and he was discharged from the hospital under stable condition with the prescribed medications. 

After two months, the vomiting was not controlled significantly so he decided to visit a gastroenterologist. The gastroenterologist recommended endoscopy and esophageal manometry, both found out to be normal but could not confirm or rule out any gastrointestinal causes for vomiting.

The patient then consulted a physician. The physician examined him and found that his blood pressure was elevated and started on antihypertensive medications. On follow-up, his blood pressure was not controlled on medications he was then referred to a cardiologist where he ordered an echocardiogram which found mild tricuspid regurgitation (TR) with pulmonary hypertension (PH). The cardiologist suspected obstructive sleep apnea (OSA) and referred him to a sleep specialist where he was diagnosed with mild OSA. The doctor suggested continuous positive airway pressure (CPAP) and stated that his vomiting might be due to sleep apnea. The patient started using CPAP. It improved patients' function and decreased the frequency of vomiting. Then the patient met another psychiatrist because the etiology of his recurrent vomiting was still inconclusive. The psychiatrist said that since he has been diagnosed with sleep apnea there is a possibility that sleep apnea will mimic depression. It would have led to chronic depression. Long-standing, untreated depression will lead to hallucinations at a later stage which is known as psychotic depression and that might have led to vomiting. The psychiatrist suggested using positive airway pressure therapy regularly and the patient subsequently reported improvement in the symptoms.

Each time when the patient approaches a physician the symptomatic treatment by a physician could bring short-lasting relief and gradually the frequency of vomiting, and associated dysfunction progresses over time. The patient was asked what triggered and decreased his vomiting episodes. He described more episodes of vomiting when he was not using CPAP or when his has disturbed sleep. The patient reported immediate vomiting when he was stressed, in a bad mood, and heard unpleasant news and when he argued with anyone. He denies obsessive or suicidal ideas.

## Discussion

Psychogenic vomiting is classified into five different types: habitual, postprandial, irregular, continuous, and self-induced. It is associated with psychiatric disorders either major depression or a conversion. The patterns of vomiting change through the clinical course. Carefully assessing the patterns and episodes of psychogenic vomiting and psychiatric disorders are vital in the diagnosis and treatment of psychogenic vomiting.

In a case series of 59 patients by Muraoka et al., admitted with a diagnosis of psychogenic vomiting reported a history of past gastrointestinal abnormalities with emotional disturbances. Not all the patients manifested purely emotional conflicts. Vomiting once occurred because of an organic or functional cause can become a habit and can be regarded as a learned form of behavior [2]. Swanson et al. reported a case series of 77 patients, where nausea was found to be the chief complaint, among which 73% were accompanied by vomiting classified as psychogenic vomiting. Hysterical neurosis was reported among 16 patients and depressive neurosis among 21 patients [8]. Rosenthal et al. in their study showed three patients with a major depression among a total of 18 patients [3]. In a systematic review of 48 studies conducted by Gupta and Simpson among patients with obstructive sleep apnea, the prevalence of major depressive disorders and post-traumatic stress disorder was found to be high [9]. 

Studies have suggested non-pharmacological treatment approaches are important as medication for psychogenic vomiting among patients with emotional disturbances. Individual and group psychotherapy, supportive and behavioral therapy, and family and marital therapy should be considered. Some studies have used a combination of supportive psychotherapy, behavioral therapy, and autogenic training as well as drugs [10]. There is a correlation between psychiatric disorders and episodes of vomiting. While planning treatment for a patient the psychological background should be carefully considered. Psychiatric disorders can change during the clinical course; therefore, the treatment plan should be modified accordingly. Psychogenic vomiting can be recognized as a unique condition or as a symptom that occurs as part of various mood, stress, and anxiety-related disorders, which can aid in early detection and proper patient management.

## Conclusions

Although the exact pathophysiology is unknown, psychogenic vomiting is primarily associated with subconscious stress. Nausea and vomiting can cause discomfort, and repeated vomiting without any organic cause can be stressful for patients and their families. A thorough medical evaluation of the patient is essential to identify any underlying organic cause of psychogenic vomiting. Psychotherapy, pharmacotherapy, aversion therapy, and behavioral interventions are needed to effectively treat patients with psychogenic vomiting.
